# Co-sparse Non-negative Matrix Factorization

**DOI:** 10.3389/fnins.2021.804554

**Published:** 2022-01-12

**Authors:** Fan Wu, Jiahui Cai, Canhong Wen, Haizhu Tan

**Affiliations:** ^1^International Institute of Finance, School of Management, University of Science and Technology of China, Hefei, China; ^2^Department of Preventive Medicine, Shantou University Medical College, Shantou, China

**Keywords:** Alzheimer's disease, co-sparse NMF, *l*_0_ constraint, structural MRI, functional MRI

## Abstract

Non-negative matrix factorization, which decomposes the input non-negative matrix into product of two non-negative matrices, has been widely used in the neuroimaging field due to its flexible interpretability with non-negativity property. Nowadays, especially in the neuroimaging field, it is common to have at least thousands of voxels while the sample size is only hundreds. The non-negative matrix factorization encounters both computational and theoretical challenge with such high-dimensional data, i.e., there is no guarantee for a sparse and part-based representation of data. To this end, we introduce a co-sparse non-negative matrix factorization method to high-dimensional data by simultaneously imposing sparsity in both two decomposed matrices. Instead of adding some sparsity induced penalty such as *l*_1_ norm, the proposed method directly controls the number of non-zero elements, which can avoid the bias issues and thus yield more accurate results. We developed an alternative primal-dual active set algorithm to derive the co-sparse estimator in a computationally efficient way. The simulation studies showed that our method achieved better performance than the state-of-art methods in detecting the basis matrix and recovering signals, especially under the high-dimensional scenario. In empirical experiments with two neuroimaging data, the proposed method successfully detected difference between Alzheimer's patients and normal person in several brain regions, which suggests that our method may be a valuable toolbox for neuroimaging studies.

## 1. Introduction

High-dimensional data structures have been available and studied in many areas including neuroimaging (Chén et al., 2018), biology (Bühlmann et al., 2014), signal processing (Shuman et al., 2013), and economics (Fan et al., 2011). Dimension reduction procedures such as principal component analysis are used to transform the data from a high-dimensional space into a low-dimensional space while possessing good interpretability.

Non-negative matrix factorization (NMF) and functional principal component analysis (FPCA) have been widely applied for dimensionality reduction in neuroimaging data over years. FPCA, an extension of multivariate principal component analysis, results in matrices with arbitrary signs using Karhunen–Loeve decomposition and the covariance matrix using the integral with respect to time. The main difference between NMF and FPCA is the non-negativity, which requires the elements of the decomposed matrices are all non-negative. Non-negativity is often more intuitive in these settings and the results are more interpretable.

In neuroimaging studies, it is more reasonable to have an NMF estimate, where the original data matrix **X** is factorized into product of two non-negative matrices, i.e., the basis matrix **W** and the coding matrix **H** (Anderson et al., 2014). A previous study successfully applies NMF to analyze the group's structural magnetic resonance imaging (MRI) and functional magnetic resonance imaging (fMRI) data to find the difference between the basis image characteristics of patients with schizophrenia and healthy controls Potluru and Calhoun (2008). Anderson et al. (2014) adopted NMF to perform unsupervised modeling of attention deficit hyperactivity disorder patients with structural MRI and fMRI, behavioral and/or phenotypic information, explaining the multimodal data of attention deficit and hyperactivity disorder through potential dimensions.

Functional connectivity (FC) effectively reveals the organization and integration of brain functions by means of describing the interaction between time series of neural activity (Mirzaei and Adeli, 2016). Decreased FC is found to cause cognition and other functions decline (Damoiseaux, 2017; Wen X. et al., 2020). The positive correlation of the resting-state FC shows that the functional synergy is existed (Fox et al., 2005). Hence, symmetry and non-negative incidence matrices are often used for fMRI functional connection (Li and Wang, 2015). The sparse representation-based methods to depict the brain activity is gradually applied in some neurophysiological study (Quiroga et al., 2005, 2008). Hence, NMF is appropriate for the processed fMRI data without the time field in our study because the functional connection matrix obtained from Pearson's correlation coefficient.

Three main types of algorithms are developed for NMF decomposition, including alternating non-negative least squares (ANLS) framework (Lawson and Hanson, 1995), multiplicative update principle (Lee and Seung, 1999), and projected gradient method (Lin, 2007). One of the first attempt in ANLS is the positive matrix factorization (Paatero and Tapper, 1994). Based on this, the general ANLS framework is proposed to solve the NMF problem, where a non-negative least squares (NNLS) technique is used to derive estimators for the two non-negative matrices. An alternative easy-to-operate and speedy method is the multiplicative update principle, which applies matrix multiplication and element-wise multiplication to update **W** and **H**, respectively. This method takes care of the non-negativity constraint in its updating equations naturally and thus return a sparse and part-based representation of input data. Although ANLS and the multiplicative update methods work well with fast calculation and speedy convergence, both types of algorithms have no theoretical guarantee for global convergence (Lin, 2007). The projected gradient, proposed by Lin (2007), is shown to enjoy desirable optimization properties including convergence rate.

When handling high-dimensional data, these classical NMF algorithms encounter challenges in both theory and algorithm (Wang et al., 2015). From the theoretical aspect, high-dimensional data might cause the non-identification issue and thus lead to the convergence problem. From the algorithmic aspect, it is difficult to recover **W** and **H** when the original data matrix **X** is high dimensional in a reasonable computational time. In high-dimensional setting, it is common to assume that there are only a small proportion of elements are non-zero, and the ℓ_*p*_-norm (0 < *p* ≤ 1) is used to restrict the number of non-zero elements. For examples, Hoyer (2002) and Hoyer (2004) used the ℓ_1_-norm due to its convexity and easy implementation. Zhang et al. (2016) proposed the coupled sparse non-negative matrix factorization model for the fusion of panchromatic and multi-spectral images via the ℓ_1/2_-norm. Based on ℓ_*p*_-norm, Dang et al. (2018) and Leng et al. (2019) introduced a smooth non-negative matrix factorization and an incremental non-negative matrix factorization, respectively. Rather than directly using the ℓ_*p*_-norm, He et al. (2017) proposed to utilize a weighted ℓ_*p*_-norm to enhance the sparsity of the abundance matrix in NMF. However, to impose sparsity on the estimated matrices, it is more straightforward to use the ℓ_0_-norm, that is, directly controlling the number of non-zero elements. In fact, the aforementioned ℓ_*p*_-norm (0 < *p* ≤ 1) is a continuous relaxation of the ℓ_0_-norm, which aims to make the implementation more easily. Rather than approximating the ℓ_0_-norm, Peharz and Pernkopf (2012) proposed to use the NNLS technique to derive the non-negative matrices, and then let the smallest elements to be zero, i.e., a hard thresholding operator to each element. This work is effective to enforce sparse structure on the matrices in a column-wise way or a row-wise way. However, it might be more reasonable to require both **W** and **H** are sparse when trying to learn useful features from a database of images.

In this paper, we propose a co-sparse non-negative matrix factorization framework to impose sparsity in both the coding matrix and the basis matrix. The co-sparsity is realized by limiting the total number of non-zero elements in both two matrices to a rather small number, which enables us to resolve the “curse of dimensionality.” This co-sparsity is similar with the work proposed by Bolte et al. (2014), where a proximal alternating linearized minimization algorithm is introduced to implement it. Yet this algorithm converges in a very slowly rate, and it is infeasible even with data of moderate size. Here, we develop a computationally efficient algorithm with block-updating rule on each matrix separably based on the primal-dual active set (PDAS) algorithm (Ito and Kunisch, 2013; Jiao et al., 2015; Wen C. et al., 2020). Due to the non-negative property of the estimation, we define a sacrifice that can discriminate non-zero and zero elements as well as satisfy the non-negativity property. Based on the synthetic experiments, the proposed algorithm not only converges in a few steps and thus is extremely fast for sparse problems, but also can accurately estimate the basis and coding matrices. We also demonstrated the effectiveness of the proposed method in application to two neuroimaging data from Alzheimer's Disease Neuroimaging Initiative (ADNI). To explore the different brain features in MRI images, a novel sparse constrained NMF method is introduced to distinguish between normal people and Alzheimer's disease (AD) patients in our study. MRI images can be linearly represented by the basis matrix **W** and the weight coefficient matrix **H**. Due to human brain is heavily connected within the same subnetworks, the connectivity between different subnetworks is sparse. Sparse NMF method can be adopted to get a sparse representation on fMRI data, where **H** represents the sparse linear superposition coefficient of the basis **W**. The interpretation of the model built by NMF is straightforward physiological because non-negativity and merging coherent functional nodes into a subnetwork.

The rest of the paper is organized as follows. In Section 2, we introduce our proposed methodology for co-sparse non-negative matrix factorization and develop an efficient iterative algorithm based on the primal-dual active set algorithm. Section 3 demonstrates comprehensive simulation studies, and Section 4 illustrates the finite sample performance of the proposal in several real data sets. Section 5 provides the conclusions and discussions.

## 2. Method and Algorithm

### 2.1. Co-sparse Non-negative Matrix Factorization

Suppose that we have a non-negative data matrix $\text{X}\in {\mathbb{R}}_{+}^{D\times N}$, where ℝ_+_ denote the non-negative real number. For a pre-specified integer *K*(≤ min{*D, N*}), the non-negative matrix factorization (NMF) aims to factorize **X** in the following way:


(1) $$\begin{array}{l} \text{X}\approx \text{W}\text{H},\text{s.t. }\text{W}\geq 0,\text{H}\geq 0, \end{array}$$

where **W** ∈ ℝ^*D* × *K*^, **H** ∈ ℝ^*K* × *N*^, and ≥ means that all elements in a matrix are non-negative. Here, **W** is called the basis matrix or dictionary and **H** is called the coding matrix. In practice, *K* is usually chosen to be much smaller than *D* and *N* in order to reduce the parameters needed to estimate.

In imaging studies, it is commonly assumed that only a small proportion of the derived coding and basis matrices contributes to the original data matrix. Under this assumption, we consider the following co-sparse non-negative matrix factorization (CSNMF) problem:


(2) $$\begin{array}{l} \begin{array}{c} \underset{\text{H}\in {\mathbb{R}}^{K\times N},\text{W}\in {\mathbb{R}}^{D\times K}}{\min}||\text{X}-\text{W H}|{|}_{F}^{2}\\ \text{ s.t. }\text{W}\geq 0,\text{H}\geq 0,\\ \;||\text{H}|{|}_{0}\leq \alpha KN,\\ \;||\text{W}|{|}_{0}\leq \beta DK, \end{array} \end{array}$$

where ||·||_*F*_ denotes the Frobenius norm, ||·||_0_ is the *l*_0_ norm counting number of non-zero elements, and α and β are two tuning parameters satisfying 0 ≤ α, β ≤ 1. The parameter α imposes sparsity in matrix **W** and corresponds to a sparse basis matrix problem (Hoyer, 2004). The parameter β restricts the number of non-zero elements in **H**, which leads to a non-negative sparse coding problem (Hoyer, 2002). If both α and β are set to be 1, then problem (Equation 2) reduces to the classical NMF problem (Lee and Seung, 1999).

### 2.2. Algorithm

The interplay between non-negative constraint and the *l*_0_-sparse constraint on both **W** and **H** poses substantial algorithmic challenges for solving the CSNMF problem in Equation 2, for which numerous state-of-art algorithms can become either inefficient or infeasible. Several algorithms are proposed to solve the problem of the least squared problem with *l*_0_ constraint. Such as, the iterative hard thresholding algorithm (Blumensath et al., 2007), the mixed integer optimization (Bertsimas et al., 2016) and the primal-dual set (PDAS) algorithm. The primal-dual active set algorithm is adopted due to its desirable theoretical property (Huang et al., 2018) and its fast speed in Wen's study (Wen C. et al., 2020). Defining a sacrifice has emerged as a key sticking point in PDAS. Sacrifice is used to define the active set and fit the sub-models with variables in active set through use of complementary primal and dual variables. For our problem, there are two constraints, which makes the problem even harder.

To address this problem, we first decouple the optimization over **W** and **H**, i.e., solve the problem (Equation 2) in a block-wise iteration by optimizing one with another one fixed. In specific, given the current estimate {**H**^(*m*)^, **W**^(*m*)^}, we solve the following two sub-problems at the (*m* + 1)th iteration:


(3) $$\begin{array}{l} {\text{H}}^{\left(m+1\right)}=\arg\underset{\text{H}\in {\mathbb{R}}^{K\times N}}{\min}||\text{X}-{\text{W}}^{\left(m\right)}\text{H}|{|}_{F}^{2},\text{ s.t. }\text{H}\geq 0,\;||\text{H}|{|}_{0}\\ \leq \alpha KN, \end{array}$$

(4) $$\begin{array}{l} {\text{W}}^{\left(m+1\right)}=\arg\underset{\text{W}\in {\mathbb{R}}^{D\times K}}{\min}||\text{X}-\text{W}{\text{H}}^{\left(m+1\right)}|{|}_{F}^{2},\text{ s.t. }\text{W}\geq 0,\;||\text{W}|{|}_{0}\\ \leq \beta DK. \end{array}$$

Given the current estimate {**H**^(*m*)^, **W**^(*m*)^}, both of the sub-problems (3) and (4) can be treated as a best subset selection problem with a non-negative constraint. Without loss of generality, we first develop a generation of the primal-dual active set (PDAS) algorithm to solve the sub-problem (3), and a similar strategy can be used to solve the sub-problem (4). The PDAS algorithm was first introduced by Ito and Kunisch (2013) and Jiao et al. (2015) for linear regression, and generalized to general convex loss function with the subset constraint by Wen C. et al. (2020). The key ingredient is how to define the sacrifice for each variable, which is used to determine the active set, i.e., the set of non-zero elements. Based on the sacrifice, the PDAS algorithm utilizes an active set updating strategy and fits the sub-models through use of complementary primal and dual variables.

To begin with, let H^*^ be the coordinate-wise minimizer of problem (3). That is, for the (*p, t*)th element of H^*^, we have


(5) $$\begin{array}{l} {\text{H}}_{pt}^{*}=\arg\min l\left({\text{H}}_{pt}\right)\text{ s.t. }{\text{H}}_{\text{pt}}\geq 0,\;||{\text{H}}_{pt}|{|}_{0}+\underset{p\neq t}{\sum }||{\text{H}}_{pt}^{*}|{|}_{0}\\ \;leq\;\alpha \;KN, \end{array}$$

where *l*(**H**_*pt*_) is the partial loss function defined by


$$\begin{array}{l} \begin{array}{c} l\left({\text{H}}_{pt}\right)=\sum_{i}\sum_{j\neq t}{\left({\text{X}}_{ij}-\sum_{k}{\text{W}}_{ik}^{\left(m\right)}{\text{H}}_{kj}^{*}\right)}^{2}+\\ \;\sum_{i}{\left({\text{X}}_{it}-\sum_{k\neq p}{\text{W}}_{ik}^{\left(m\right)}{\text{H}}_{kt}^{*}-{\text{W}}_{ip}^{\left(m\right)}{\text{H}}_{pt}\right)}^{2}. \end{array} \end{array}$$

Note that *l*(**H**_*pt*_) is a quadratic function of **H**_*pt*_. Let h^*^ be the optimizer of *l*(**H**_*pt*_) by ignoring the constraint in Equation (5). Following Wen C. et al. (2020), we consider the sacrifice of the (*p, t*)th element if we switch **H**_*pt*_ from h^*^ to 0 as


$$l\left(0\right)-l\left(h^{*}\right)=||{\text{W}}_{\cdot p}^{\left(m\right)}|{|}_{2}^{2}{\left({\text{H}}_{pt}^{*}\right)}^{2},$$

where $∥{\text{W}}_{.p}^{\left(m\right)}{∥}_{2}^{2}$ is the sum of squares of the elements in the *p*th column of the matrix **W**^(*m*)^. Since we need to guarantee the non-negative property for **H**_*pt*_, we modify the definition of the sacrifice as


(6) $$\Delta_{pt}=\{\begin{array}{ll} {\left\|W_{\cdot p}^{\left(m\right)}\right\|}_{2}^{2}{\left(H_{pt}^{*}\right)}^{2}, & \text{ if }H_{pt}^{*}\geq 0\\ 0, & \text{ otherwise. } \end{array}$$

The sacrifice measures the importance of elements in H^*^, which can be used to screen out the unimportant elements. That is, among all the elements in H^*^, we may enforce those ${\text{H}}_{pt}^{*}$ to zero if they contribute the least total sacrifice to the overall loss. To realize this, calculate sacrifice for each ${\text{H}}_{pt}^{*}$ by Equation (6) and rearrange them by decreasing order:


$$\Delta_{\left[1\right]}\geq \Delta_{\left[2\right]}\geq ...\geq \Delta_{\left[\alpha KN\right]},$$

where [·] means the rearrangement order by sacrifice.

Then, define the active set for **H**, $A=\left\{\left(p,t\right)|{\text{H}}_{pt}>0,p=1,\ldots ,D,t=1,\ldots ,K\right\}$ with cardinality as α*KN*, and inactive set $I=A^{c}$ with cardinality as (1−α)*KN*. Then the active and inactive sets of **H** can be determined by {Δ_*pt*_, *p* = 1, …, *D, t* = 1, …, *K*}. In specific, at the *m*th iteration with current solution {**H**^(*m*)^, **W**^(*m*)^}, we can estimate A and I by


$$\begin{array}{l} A^{\left(m\right)}=\left\{\left(p,t\right)|\Delta_{pt}^{\left(m\right)}\geq \Delta_{\left[\alpha KN\right]}^{\left(m\right)}\right\},\\ I^{\left(m\right)}=\left\{\left(p,t\right)|\Delta_{pt}^{\left(m\right)}<\Delta_{\left[\alpha KN\right]}^{\left(m\right)}\right\}, \end{array}$$

where $\Delta_{pt}^{\left(m\right)}$ is an estimate of Δ_*pt*_ by replacing ${\text{H}}_{pt}^{*}$ in Equation (6) by ${\text{H}}_{pt}^{\left(m\right)}$.

After the determination of active set, we can update the final estimate of **H** by restricting the non-zero elements, i.e., we may estimate each column of the [α*KN*]-non-zero coding matrix by the NNLS algorithm (Lawson and Hanson, 1995):


(7) $$\begin{array}{l} {\text{h}}_{j}^{\left(m+1\right)}=\arg\min||{\text{x}}_{j}-{\text{W}}^{\left(m\right)}\text{h}|{|}_{F},\;\text{s.t. }{\text{h}}_{I^{\left(m\right)}}=0\text{ and }{\text{h}}_{A^{\left(m\right)}}\geq 0. \end{array}$$

Then the final estimate of coding matrix is ${\text{H}}^{\left(m+1\right)}=\left({\text{h}}_{1}^{\left(m+1\right)},\ldots ,{\text{h}}_{N}^{\left(m+1\right)}\right)$. Similar strategy can be applied for solving the sub-problem (4) and details are omitted here for concise. We summary the above discussion in the alternative primal-dual active set (APDAS) algorithm as follows.

** Remark 1**. *To speedy the algorithm and get a smaller reconstruction error, we adopt the following strategy to reduce the computational burden. When α is less than β, we update matrix **H** first, and then update **W**. When β is less than α, the order of update is reversed*.

** Remark 2**. *To ensure the recognizability of the result, it is necessary to rescale each column of **W** to unity after **W** is updated. If the update order is **H** first and then **W**, **W** does not need to be unitized in the last iteration*.

**Algorithm 1:** Alternative primal-dual active set (APDAS) algorithm.

**Require:** Data $\text{X}\in {\mathbb{R}}_{+}^{D\times N}$, sparse levels (α, β), the maximum number of iterations *m*_max_, iteration stopping threshold ε.
**Ensure:** $\left\{\hat{\text{W}},\hat{\text{H}}\right\}=\left\{{\text{W}}^{\left(m+1\right)},{\text{H}}^{\left(m+1\right)}\right\}$.
1: **Initialization:** Initialize **W**^(0)^ and **H**^(0)^ with elements from uniform distribution *U*(0, 1).
2: **for** *m* = 1, 2, …, *m*_max_ **do**
3: **if** α ≠ 1 **then**.
4: • Calculate the sacrifice $\Delta_{pt}^{\left(m\right)}$ by replacing ${\text{H}}_{pt}^{*}$ in Equation (6) by ${\text{H}}_{pt}^{\left(m\right)}$.
5: Update active set and inactive set by
6:
$$\begin{array}{l} A_{\text{H}}^{\left(m\right)}=\left\{\left(p,t\right)|\Delta_{pt}^{\left(m\right)}\geq \Delta_{\left[\alpha KN\right]}^{\left(m\right)}\right\},\\ I_{\text{H}}^{\left(m\right)}=\left\{\left(p,t\right)|\Delta_{pt}^{\left(m\right)}\leq \Delta_{\left[\alpha KN\right]}^{\left(m\right)}\right\}. \end{array}$$
7: • Update **H**^(*m*)^ by ${\text{H}}^{\left(m+1\right)}=\left({\text{h}}_{1}^{\left(m+1\right)},\ldots ,{\text{h}}_{N}^{\left(m+1\right)}\right)$, where ${\text{h}}_{j}^{\left(m+1\right)}$ is computed from (7).
8: **end if**
9: **if** β ≠ 1 **then**
10: • Calculate the sacrifice $\Delta_{pt}^{\left(m\right)}$ in a similar way with Equation (6).
11: Update active set and inactive set by
12:
$$\begin{array}{l} A_{\text{W}}^{\left(m\right)}=\left\{\left(p,t\right)|\Delta_{pt}^{\left(m\right)}\geq \Delta_{\left[\beta DK\right]}^{\left(m\right)}\right\},\\ I_{\text{W}}^{\left(m\right)}=\left\{\left(p,t\right)|\Delta_{pt}^{\left(m\right)}\leq \Delta_{\left[\beta DK\right]}^{\left(m\right)}\right\}. \end{array}$$
13: • Update **W**^(*m*)^ by ${\left(W^{\left(m+1\right)}\right)}^{\text{T}}=({\left(w^{\left(m+1\right)}\right)}_{1}^{\text{T}},\ldots ,{\left(w^{\left(m+1\right)}\right)}_{D}^{\text{T}}$), where ${\left({\text{w}}^{\left(m+1\right)}\right)}_{j}^{\text{T}}$ is computed similar to (7).
14: **end if**
15: Column normalization of **W**^(*m*)^: ${\text{W}}_{ik}^{\left(m\right)}={\text{W}}_{ik}^{\left(m\right)}/\underset{i}{\sum }{\text{W}}_{ik}^{\left(m\right)}$.
16: **if** $\frac{||\text{X}-{\text{W}}^{\left(m+1\right)}{\text{H}}^{\left(m+1\right)}|{|}_{F}}{||\text{X}|{|}_{F}}\leq \varepsilon$ **then** stop.
17: **else** *m* = *m* + 1 and return to steps 2−17.
18: **end if**
19: **end for**

** Remark 3**. *To increase the estimation accuracy, we add a re-calibration step before the above procedure, i.e., we re-estimate the current solution by a fast combinational NNLS (FC-NNLS) algorithm (Van Benthem and Keenan, 2004). FC-NNLS can be used instead of NNLS to more conveniently and efficiently solve large-scale non-negative constrained least squares problems*.

**Figure 1:**
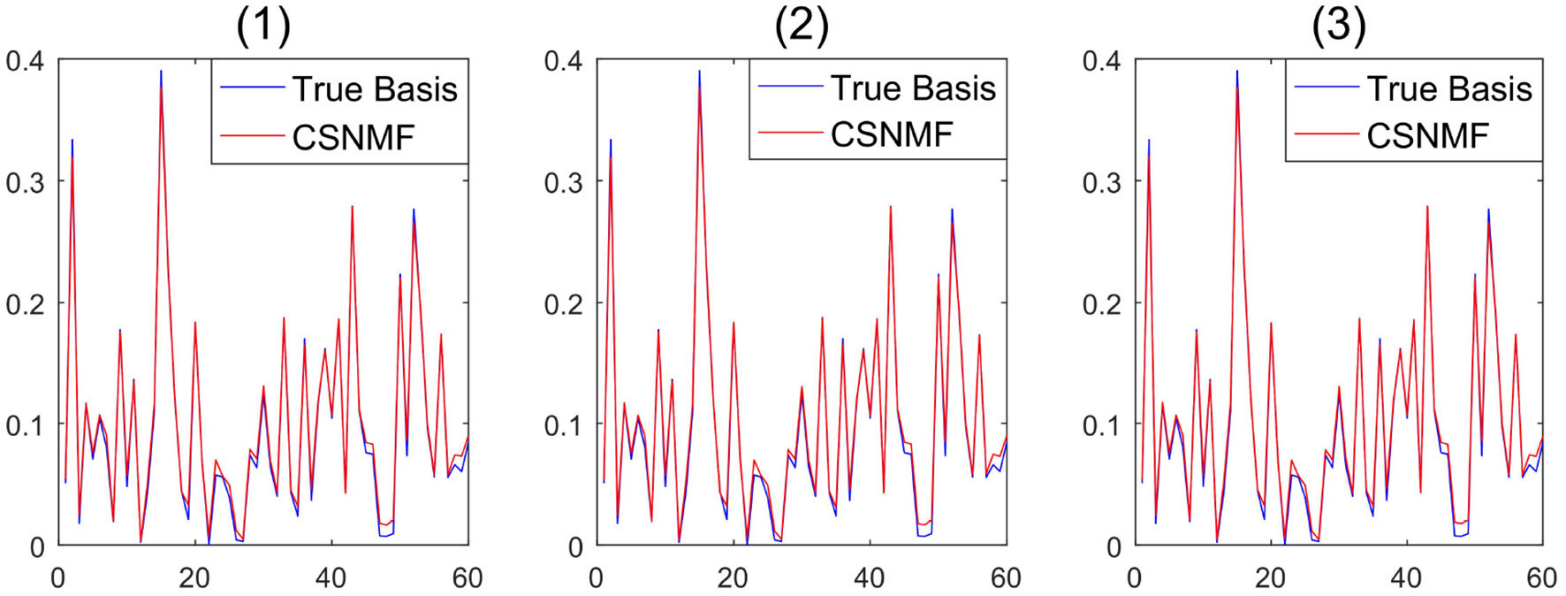
Basis curve restored by three initialization methods: (1) randomly generated matrices; (2) random matrices with its elements being sampled from the original observation matrix **X**; (3) matrices generated by singular vector decomposition of the original observation matrix **X**.

**Figure 2:**
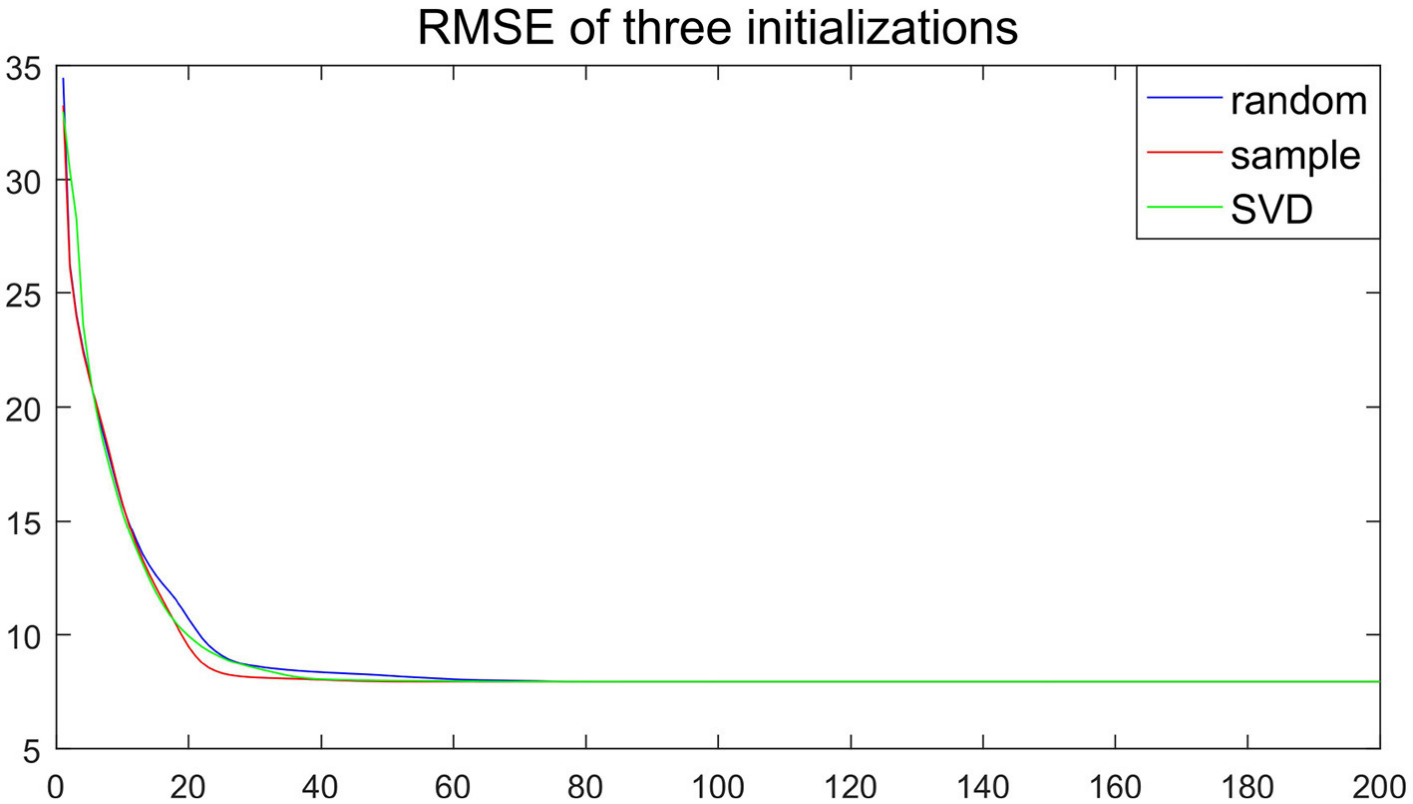
Root mean squared error (RMSE) curve of three initialization methods.

** Remark 4**. *Compared to classical NMF problem, NMF problem with sparse inducing constraint effectively controlled the non-uniqueness problem of **W** and **H** (Eggert and Korner, 2004). To demonstrate the stability of the proposed algorithm, we take SNR=20, m=120 in simulation 1, and consider three initialization: (1) randomly generated matrices; (2) random matrices with its elements being sampled from the original observation matrix **X**; (3) matrices generated by singular vector decomposition of the original observation matrix **X**. In Figure 1, the output basis matrix $\hat{\text{W}}$ obtained from these three initialization strategies shows that the estimated basis matrices are almost similar and close to the true basis matrix. Figure 2 depicts that the root mean squared error (RMSE) varies with the number of iterations for indicating that proposed CSNMF converge rate under the second strategy is the fastest. Consequently, CSNMF is certified to be stable and its convergence rate is influenced by difference of initial values*.

**Figure 3:**
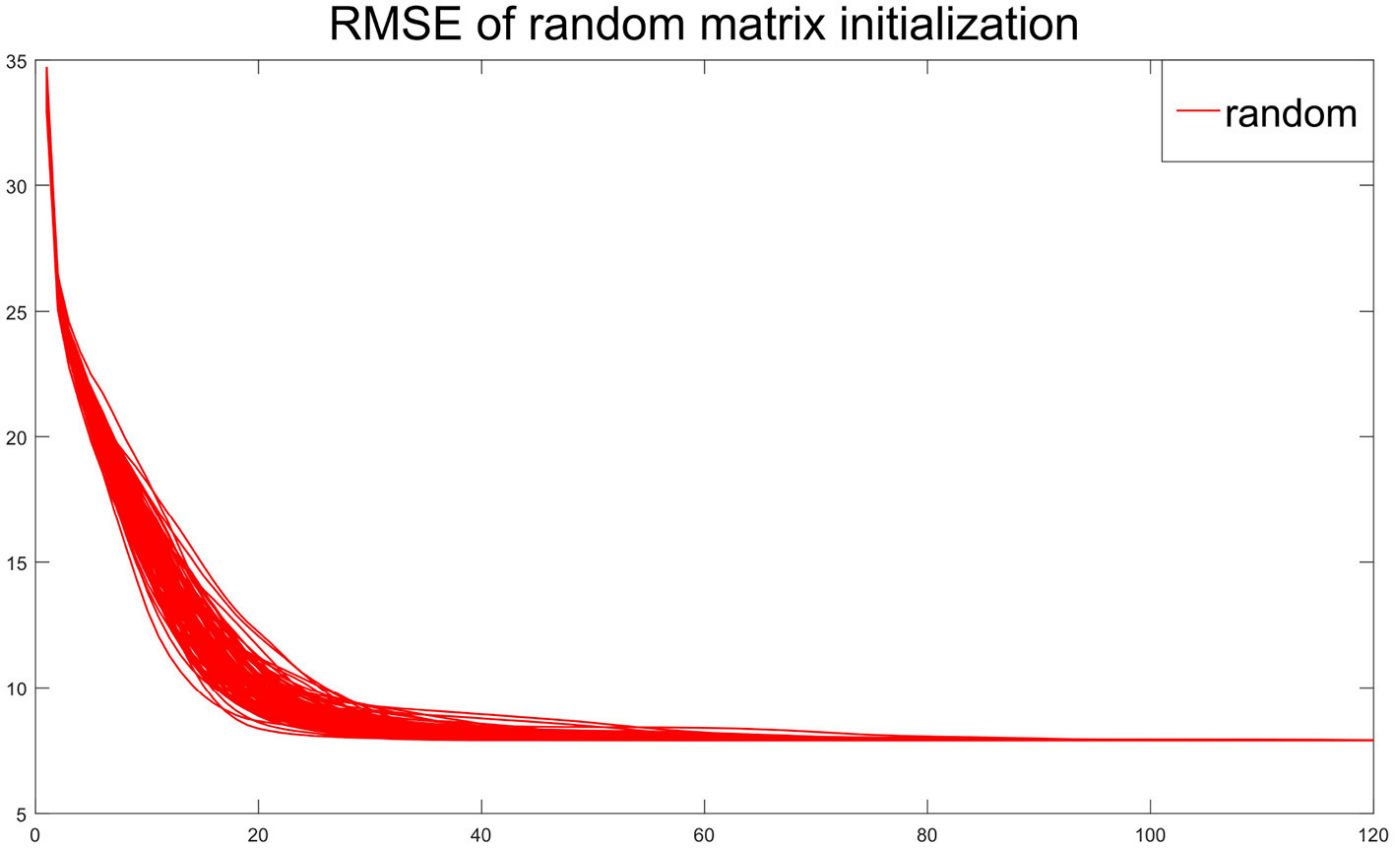
Root mean squared error (RMSE) curve for 100 times with initial value of random matrix.

** Remark 5**. *Figure 3 depicts the RMSE of the estimator vs. the iteration times with the initial value of the random matrix in 100 independent replications under the experimental conditions of Remark 4. Although the initial value is different, the RMSE decline trajectory is different, Figure 3 shows about 20 iterations get close to convergence. The result of each experiment converged to a stable value after 80 iterations. The similar phenomena in other simulation settings are observed. Hence, Figure 3 suggests that the convergence result of CSNMF is very stable*.

### 2.3. Comparison With Existing Methods

The proposed framework in Equation (2) can be regarded as a comprehensive sparse learning in the non-negative matrix factorization problem for the high-dimensional data. Another method proposed by Bolte et al. (2014) also considered the problem (Equation 2) with [α*KN*] and [β*DK*] being replaced by integers *s*_1_ and *s*_2_, but a totally different algorithm is proposed for solving it. They developed a proximal alternating linearized minimization (PALM) algorithm, where a proximal map formula is used to eliminate the zero elements.

There are significant differences lying behind between our proposal and the PALM algorithm. First, the proximal map formula is actually a truncating step with an additional hard thresholding rule to make the input matrix to be both non-negative and sparse. This formula is used after the estimation to simply make the constraints to be satisfied. Yet in our proposal, the non-negativity is embedded into the determination of active and inactive sets, and the sparsity is determined by the sacrifice, which is related to the necessary condition of the problem (Equation 2). Second, the PALM algorithm use a pseudo-Newton updating rule to update the current estimates for both **W** and **H** regardless of the constraints. To make the algorithm to be efficient, we need to determine a suitable stepwise, which would hinder its use in practice. In contrast, there is no tuning parameter in our proposal since the non-zero entries are estimated by the NNLS algorithm. Third, due to the pseudo-Newton updating rule, the PALM algorithm converges in a very slowly rate, usually in hundreds even for a low-dimensional data. Our CSNMF converges in a few steps and is extremely fast for very sparse problem. This is because that after the active set is determined, we derive the optimal estimate for the non-zero elements rather than updating them with a pseudo-Newton step.

When α = 1 or β = 1, the problem (Equation 2) reduces to the sparse estimation on **W** or **H**, which is similar to those considered in Peharz and Pernkopf (2012). In specific, they consider the following problems:


(8) $$\begin{array}{l} \begin{array}{c} \underset{\text{W},\text{H}}{\min}||\text{X}-\text{W H}|{|}_{F}^{2}\\ \text{s.t. }\text{W}\geq 0,\text{H}\geq 0,\\ ||{\text{h}}_{i}|{|}_{0}\leq \alpha K,\;i=1,\ldots ,N, \end{array} \end{array}$$

and


(9) $$\begin{array}{l} \begin{array}{c} \underset{\text{W},\text{H}}{\min}||\text{X}-\text{W H}|{|}_{F}^{2}\\ \text{s.t. }\text{W}\geq 0,\text{H}\geq 0,\\ ||{\text{w}}_{j}|{|}_{0}\leq \beta D,\;j=1,\ldots ,K. \end{array} \end{array}$$

The formal problem is regarded as the NMF ℓ_0_-H problem and the latter one is named as the NMF ℓ_0_-W problem. Although they are closely related to our problem, there are still substantial differences between them. While the problem (8) or (9) restricts the number of non-zero elements within each column, we impose sparsity on the whole matrix and relax the sparsity in each column. Thus, the optimum of (8) or (9) is larger than those of Equation (2) with β = 1 or α = 1. When the columns have comparable number of non-zero entries, these algorithms achieve similar results. However, when the columns have unbalanced number of non-zero entries, the algorithms proposed by Peharz and Pernkopf (2012) cannot converge to a solution, yet our proposal could still derive an optimal solution.

## 3. Synthetic Experiment

In this section, we use synthetic data to verify the effectiveness of the CSNMF algorithm in three aspects. The first two simulation studies restrict the non-zero elements in the coding matrix **H** and the basis matrix **W**, respectively. The third simulation study illustrates the control of the number of non-zero elements in both **H** and **W**. To be compared, we also consider the NMF *l*_0_-H or NMF *l*_0_-W (Peharz and Pernkopf, 2012) and PALM-SNMF (Bolte et al., 2014) algorithms.

To evaluate the finite-sample performance of different methods, we consider the following measurements. The first one is the signal-to-noise ratio (SNR), which is defined by


$$\text{SNR}=10\log_{10}\frac{||\text{X}|{|}_{F}^{2}}{||\text{X}-\hat{\text{W}}\hat{\text{H}}|{|}_{F}^{2}},$$

where **X** is the original data matrix, and $\hat{\text{W}}$ and $\hat{\text{H}}$ are the sparse NMF estimators. The SNR is used to evaluate the reconstruction accuracy, with the larger SNR value indicates better performance. The second measure is the basis distance defined by $||\hat{\text{W}}-\text{W}|{|}_{F}$. It measures the estimation accuracy of the basis matrix and the smaller the better.

### 3.1. Simulation I

The first simulation study considers a sparse basis matrix **H** with sparsity level α = 0.2. We first determine the position of non-zero entries by a discrete uniform distribution, and then fill them with the absolute values of random variables from *N*(0, 1). For the basis matrix **W**, we draw random variables from the standard Gaussian distribution and take its absolute value for each element. Then, each column of **W** is normalized to be unit length so that it can be treated as a basis vector. We fix the number of basis vector *D* to be 60, *N* = 1, 000, *m* = 300, and let *K* chosen from {40, 60, 80}. Finally, the data matrix is generated by using


X=WH+E,

where **E** consists of uniformly and positive random variables. In specific, the noise **E** is generated by the following equation:


$$\begin{array}{l} \text{E}={\text{E}}_{0}\text{ diag}\left(\sqrt{\frac{{\text{S}}_{WH}}{10^{s/10}{\text{S}}_{E_{0}}}}\right), \end{array}$$

where **E**_0_ is drawn from Uniform distribution *U*(0, 1), and **S**_*WH*_ and **S**_*E*_0__ are the summation of columns in matrix **WH** and **E**_**0**_, respectively. The *s* represents the true SNR and is chosen from {5, 10, …, 50}. The above procedure is replicated 10 times for each combination of SNR and *K*. Although, Bolte et al. (2014) recommends setting the gradient descent step size greater than 1, we have found that a better fitting result can be obtained by taking 0.8 in experiments. Since the gradient descent is very slow, to ensure convergence, the number of executions is set to *m* × 50. For comparison, the Basis Matrix Update step in NMFℓ_0_-H is executed for once.

**Figure 4:**
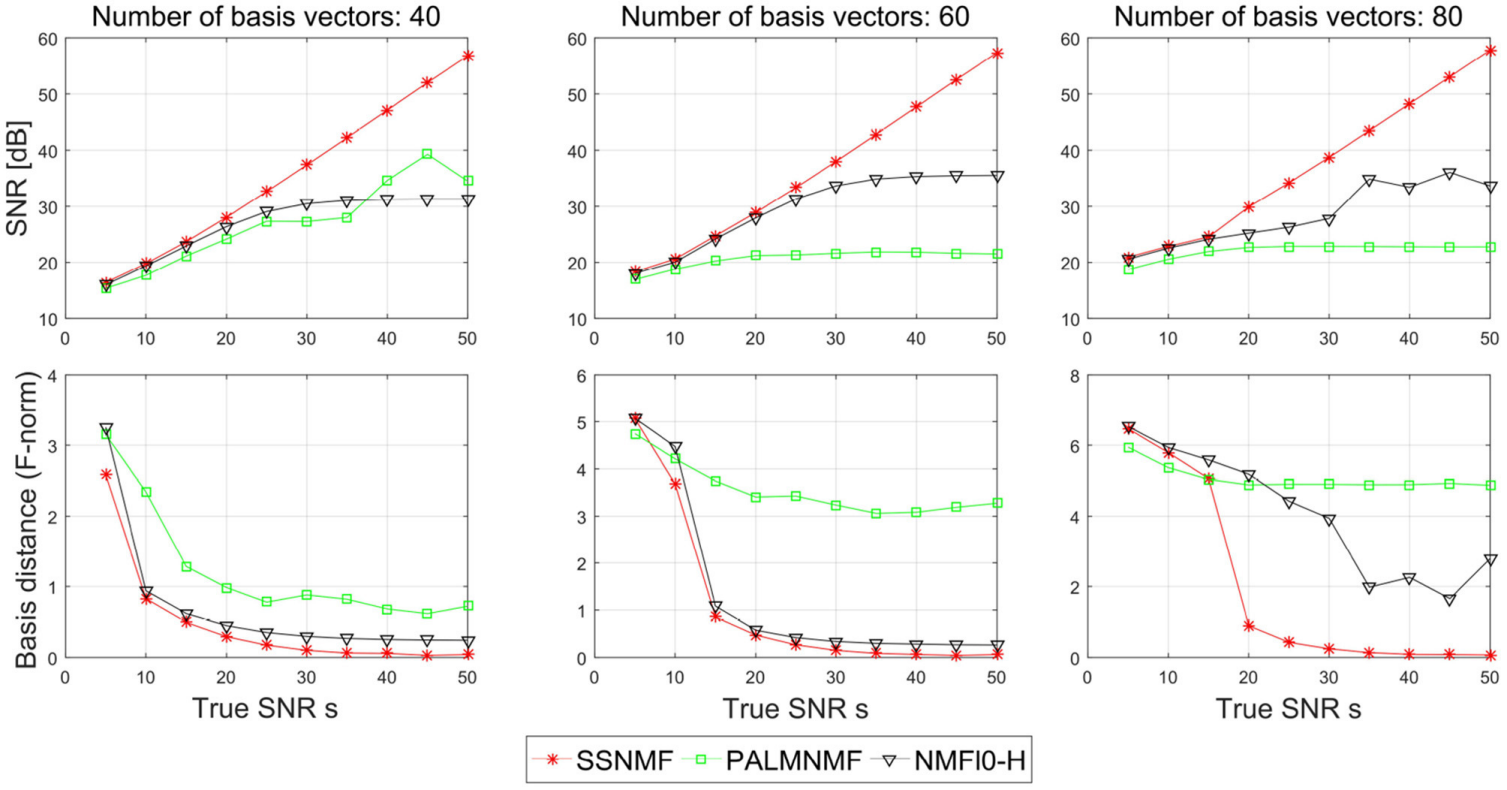
Plots of the signal-to-noise ratio (SNR) and basis distance vs. the true SNR *s* for different algorithms in simulation I.

Figure 4 shows the SNR and the basis distance vs. *s* for different methods. Compared with NMFℓ_0_-H and PALM-SNMF, our proposed CSNMF has consistently better performance with higher reconstruction quality and smaller basis distance. In terms of SNR, both NMFℓ_0_-H and PALM-SNMF have satisfied performance when the true SNR is small, yet they cannot improve the performance as the true SNR increase when the true SNR is large. In contrast, the estimated SNR of CSNMF is approximately a linear function of the true SNR, which indicates that the CSNMF estimator is able to recover the true signal and retain all important information in **X**. Moreover, as the true SNR *s* increase, while the basis distance of PALM-SNMF preserves a substantial gap from zero, the basis distance for the other two algorithms approach to zero when the number of dictionary *K* is small. When *K* = 80 and the true SNR is high, whereas the basis distance NMFℓ_0_-H fluctuates around 2, the gap between the estimated and true basis for our proposal narrows almost to vanishing point. This suggests that the basis matrix can still be identified well by CSNMF even when the number of dictionary *K* is large.

**Figure 5:**
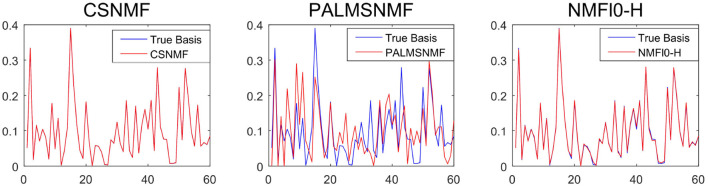
The estimated basis vector curve obtained by co-sparse non-negative matrix factorization (CSNMF), PALM-SNMF and non-negative matrix factorization (NMF)ℓ_0_-H as well as the true basis vector when *K* = 40 and *s* = 50.

To provide further insights into the basis estimation, we plot one of the estimated basis vectors from the three approaches with *K* = 40 and SNR = 50 as well as the true basis vector for reference in Figure 5. It can be seen that CSNMF has best performance, which can almost recover the true basis vector, while the other two methods can not.

### 3.2. Simulation II

**Figure 6:**
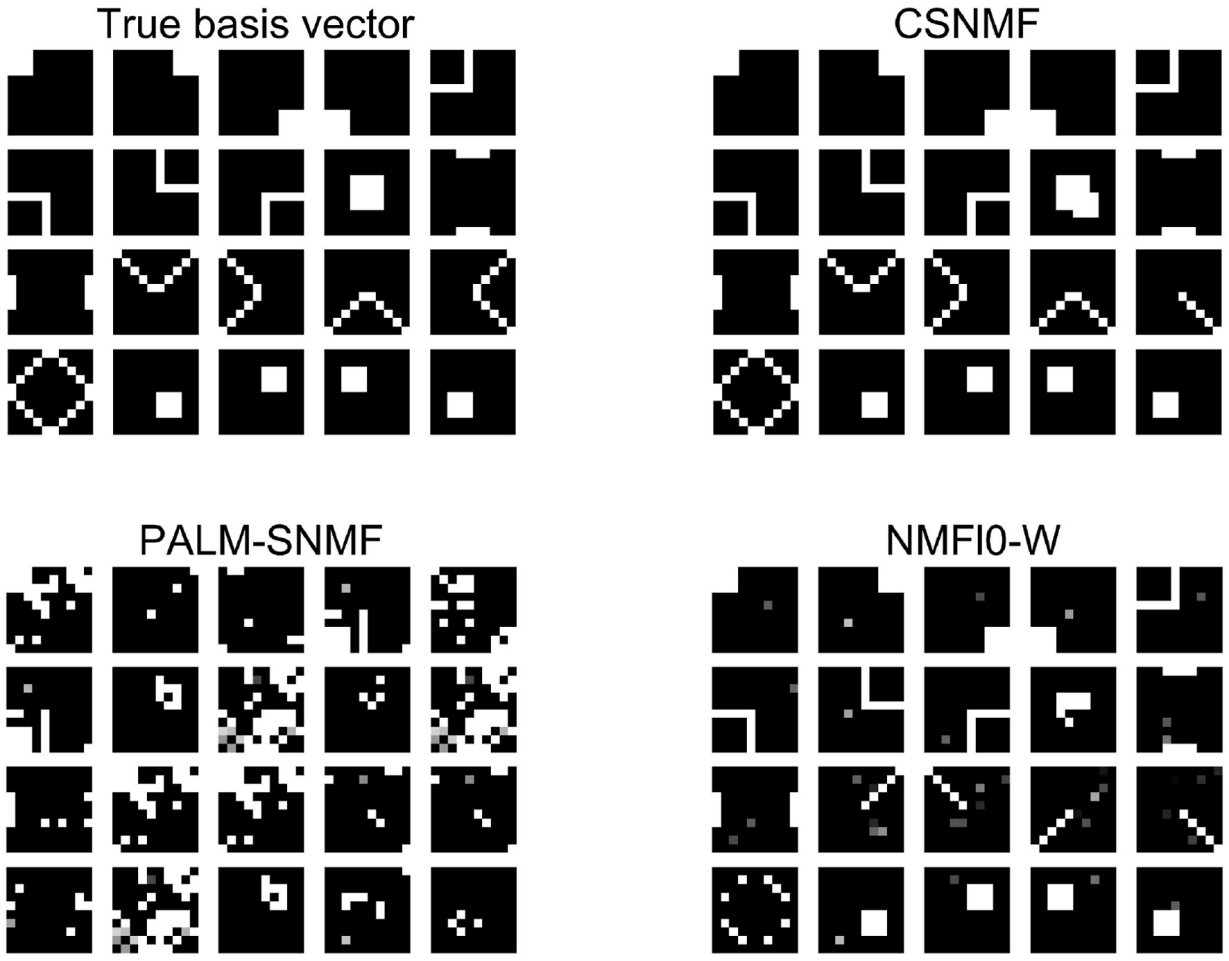
The result of *l*_0_ sparsity control on **W**. The first image on the left is the base graphics that forms the data matrix **X**. The next three pictures are the estimated basis pattern via the three algorithms.

In the second simulation study, we consider 20 basis vectors constructed by different shapes of size 10 × 10, as shown in the top left panel of Figure 6. In each shape, the value of white pixels is 1 and that of black pixels is 0. Thus, there are 10% elements in **W** are non-zero. Similarly, we generate the each element of the coding matrices **H** by taking absolute value of independent standard Gaussian noise. The data matrix is generated from


X=WH+E,

where **E** is generated in the same way as Simulation I with *s* = 20. Here, we set *K* = 20 and *N* = 100. We executed our algorithm and NMF *l*_0_-W for 80 iterations. We executed PALM-SNMF for 4000 iterations to ensure convergence. We did not perform more iterations because the NMF *l*_0_-W algorithm started to fail to converge after running 80 times, and the number of non-zero elements and the SNR decreased rapidly.

Figure 6 shows the output estimated basis for each method, from which we can see that the basis matrix found by CSNMF is the closest to the true basis matrix. In particular, CSNMF recover almost all the real basis vectors with only 2 patterns deviated from the real basis. In contrast, NMF *l*_0_-W roughly recover the pattern of the basis vectors, but many of them cannot be distinguished completely. The patterns found by PALM-SNMF is basically different from the true ones. In total, when the matrix **X** has a sparse structure, CSNMF has better ability to find the true basis vector. AS for SNR, the value of CSNMF is 23.1664, which is greater than 4.7088 and 12.3383 of NMF *l*_0_-W.

### 3.3. Simulation III

In the third simulation study, both **W** and **H** are assumed to be sparse. In specific, let α be chosen from {0.2, 0.4, 0.6} and β be chosen from {0.2, 0.4, 0.6}. For both two matrices, the positions of the non-zero elements are randomly from a discrete uniform distribution, and the non-zero elements are filled with absolute value of random variables from chi-square distribution with freedom 1. To facilitate the identification issue in basis matrix, we normalize each column of **W** to be unit length. We fix the number of dimension *D* to be 300, the sample size *N* to be 300, m=100, and *K*=60. We consider the true SNR *s* being chosen from {5, 10, …, 50}. The error term **E** is generated in the same way as in simulation I. Finally, we generate **X** by the equation **X** = **WH** + **E**. For each combination of s, α and β, the experiment is repeated 10 times independently and the average value was taken.

**Figure 7:**
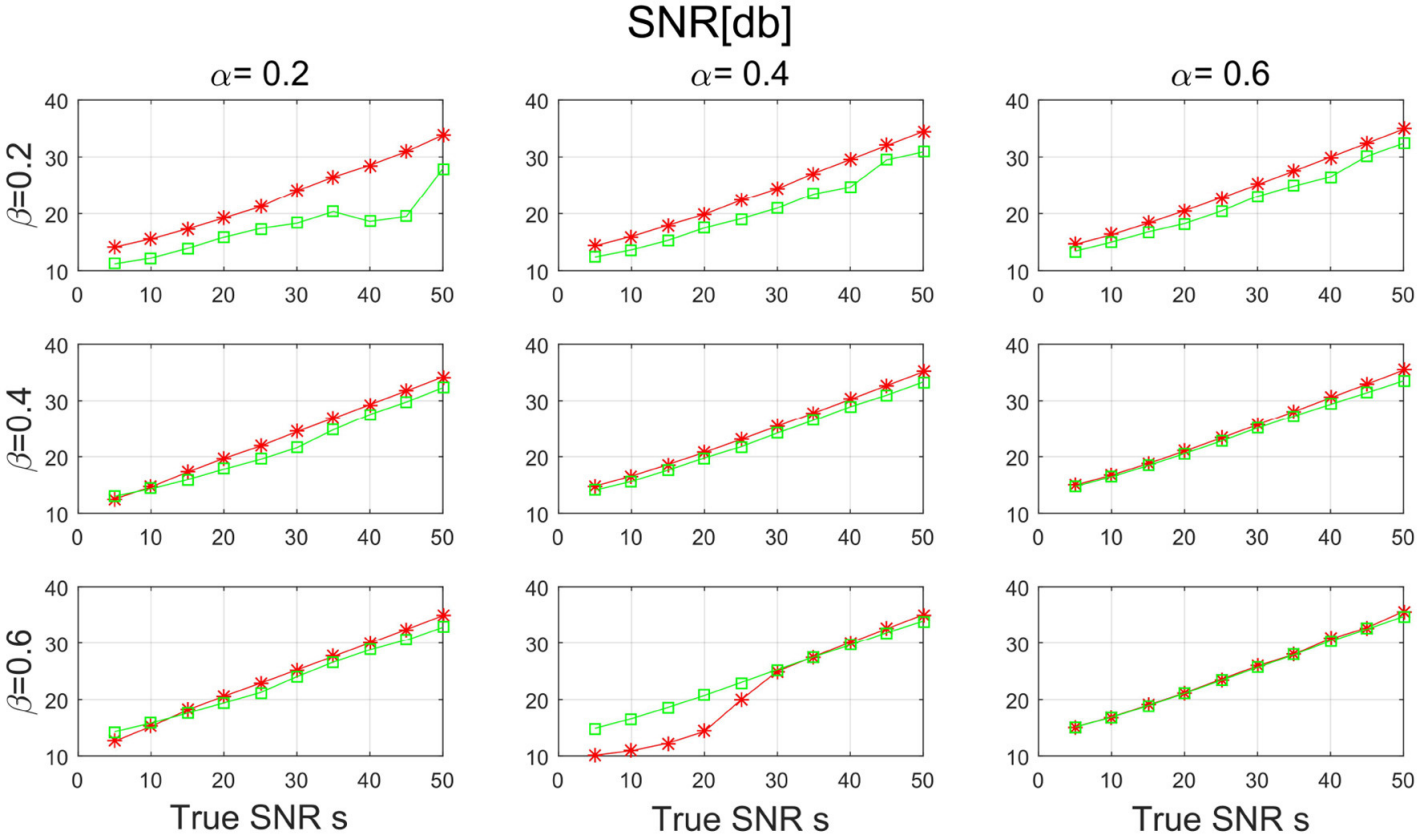
The result of signal-to-noise ratio (SNR) with sparsity level α and β from {0.2, 0.4, 0.6} for PALM-SNMF and co-sparse non-negative matrix factorization (CSNMF).

**Figure 8:**
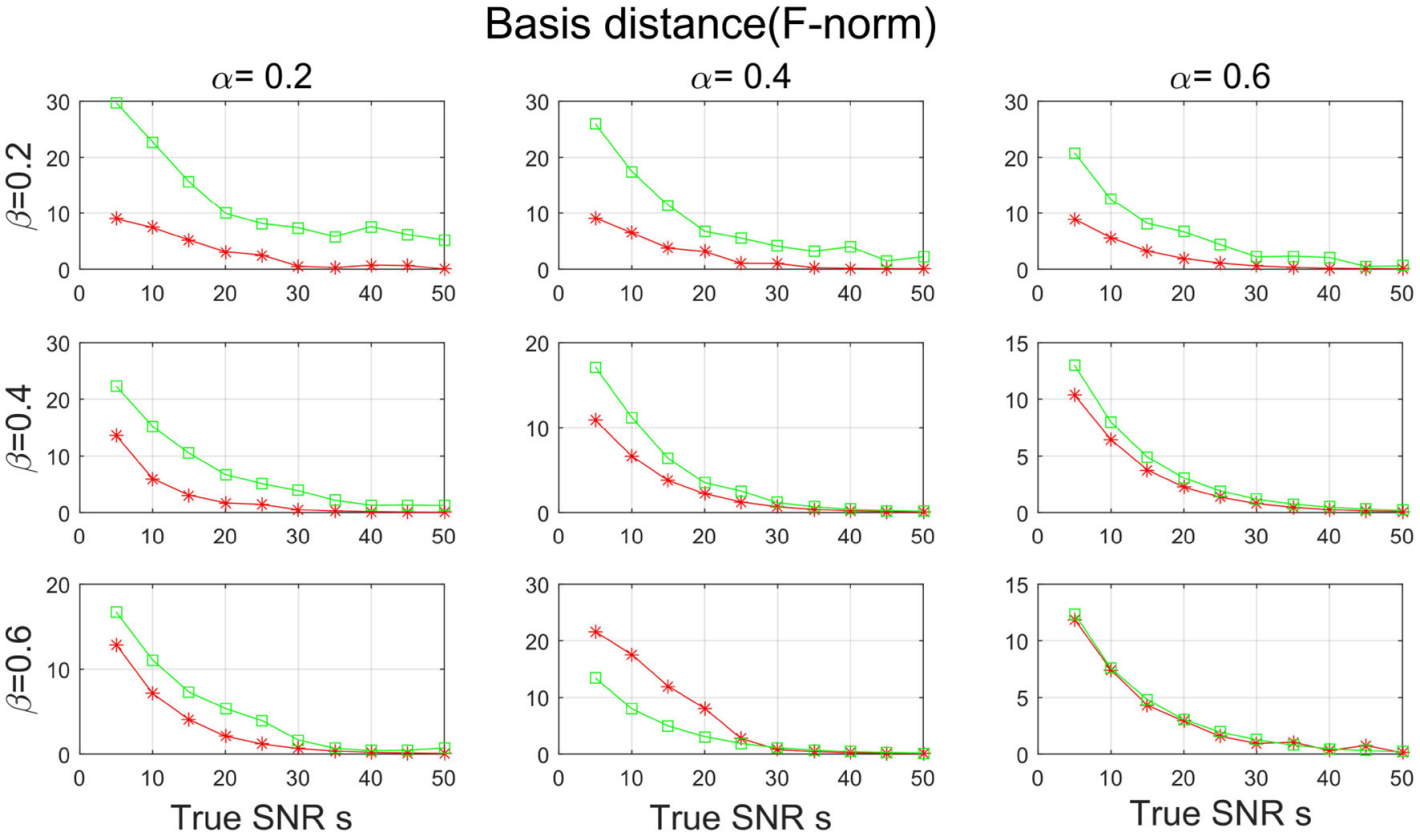
The result of basis distance with sparsity level α and β from {0.2, 0.4, 0.6} for PALM-SNMF and co-sparse non-negative matrix factorization (CSNMF).

Figures 7, 8 show the SNR and the basis distance vs. *s* for different values of α and β. Compared with PALM-SNMF, CSNMF has obvious advantages in terms of SNR and basis distance when the sparsity level α or β is small. Particularly, the basis distance of PALM-SNMF is closer to 0 when α = 0.2, β = 0.2 and *s* = 5, which means that in the case of high noise, sparse basis space and weight coefficient, our algorithm can obtain results that are closer to the real basis space and have a higher SNR. Furthermore, our algorithm can still figure out the true basis vectors in data with high noise signal. For example, when α = 0.2 and β = 0.4, the basis distance of CSNMF is close to 0 when *s* = 25. The estimated SNR of CSNMF is almost a linear function of s, indicating that it can better retain the information of the data. With the increase of α and β, it becomes harder to recover the true basis vectors. Since **W** and **H** become less sparse, the decomposition results of the two algorithms are similar.

## 4. Empirical Experiment

In this section, we assess the performance of our proposal in two data sets from neuroimaging studies in comparison with the estimators from NMF *l*_0_-H (Peharz and Pernkopf, 2012) and PALM-SNMF (Bolte et al., 2014). In the first data set, we explore the advantages of the proposed CSNMF in terms of local feature representation, convergence, and reconstruction error. The second data set is used to show the differences in brain FC between people with AD patients and cognitively normal (CN) people. We measure the reconstruction accuracy in terms of the root mean square error (RMSE):


$$\text{RMSE}=||\text{X}-\hat{\text{W}}\hat{\text{H}}|{|}_{F},$$

where $\hat{\text{W}}$ and $\hat{\text{H}}$ are the sparse NMF estimators.

The two data sets are provided by the Alzheimer's Disease Neuroimaging Initiative (ADNI) database (http://adni.loni.usc.edu/). ADNI was launched in 2003 by the National Institute on Aging, the National Institute of Biomedical Imaging and Bioengineering, the Food and Drug Administration, private pharmaceutical companies, and non-profit organizations as a $60 million and 5-year public-private partnership. The primary goal of ADNI was to test whether serial MRI, PET, and other biological markers are useful in clinical trials of mild cognitive impairment (MCI) and early AD. The determination of sensitive and specific markers of very early AD progression is intended to aid researchers and clinicians to develop new treatments and monitor their effectiveness and estimate the time and cost of clinical trials. ADNI subjects aged 55–90 years old and from over 50 sites across the USA and Canada participated in the research; more detailed information is available at www.adni-info.org.

### 4.1. MRI Data

**Figure 9:**
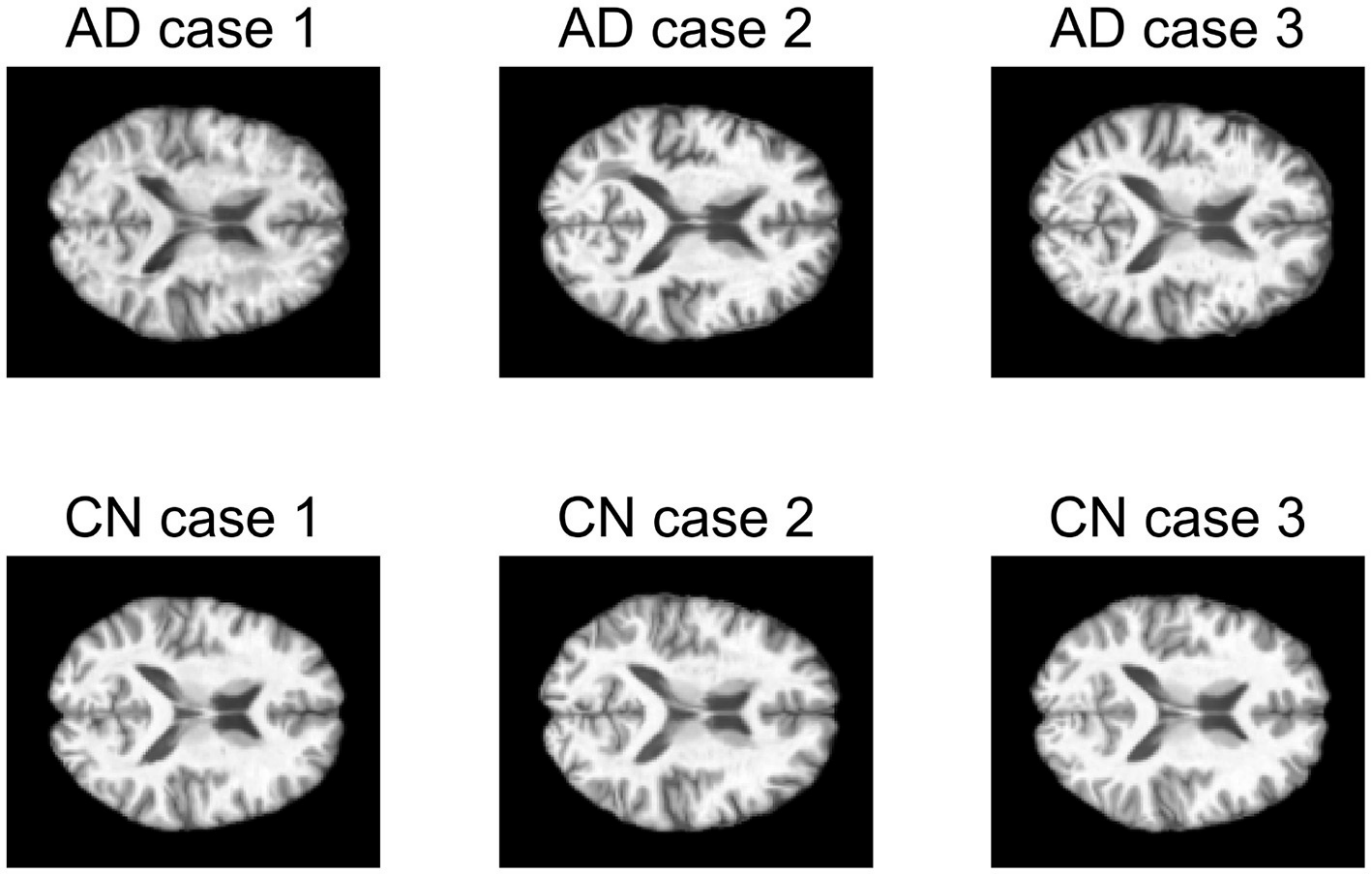
Examples of magnetic resonance imaging (MRI) images of AD patients and CN people.

The first data set consists of structural magnetic resonance imaging (MRI) scans. In this study, 249 MRI scans obtained from ADNI database were used. The scans from 107 AD patients and 142 CN people were performed on a 1.5T MRI scanners with some individual protocols. Here, we try to decompose the MRI image for AD patients and CN people individually, and would like to see the difference between these two groups of population. As a demonstration, we selected the central image for each subject, i.e., the 60-th image of size 121 × 145, and then vectorized it. Figure 9 shows some examples for AD patients and CN. Therefore, we have two data matrices **X**_*AD*_ and **X**_*CN*_ with size of 17, 545 × 107 and 17, 545 × 142, respectively.

We set the number of basis vectors to be *K* = 25 and the sparsity level of coding matrix to be α = 1. To investigate the influence of sparsity of basis matrix, we vary it from 0.04 to 0.08 with a step size 0.02. For each scenario, we replicate the above experiment for 10 times. In each replication, we record the RMSE for each algorithm, and the final RMSE are averaged over different choices of *K* and different replications. Since PALM-SNMF needs more iterations to converge, both the CSNMF and NMF ℓ_0_-W algorithms run 40 iterations and PALM-SNMF are executed for 2,000 iterations. To be fair, we compare the results of CSNMF and NMF ℓ_0_-W in the *i*th iteration to those of PALM-SNMF in the 50 × *i* iteration, *i* = 1, …, 40.

**Figure 10:**
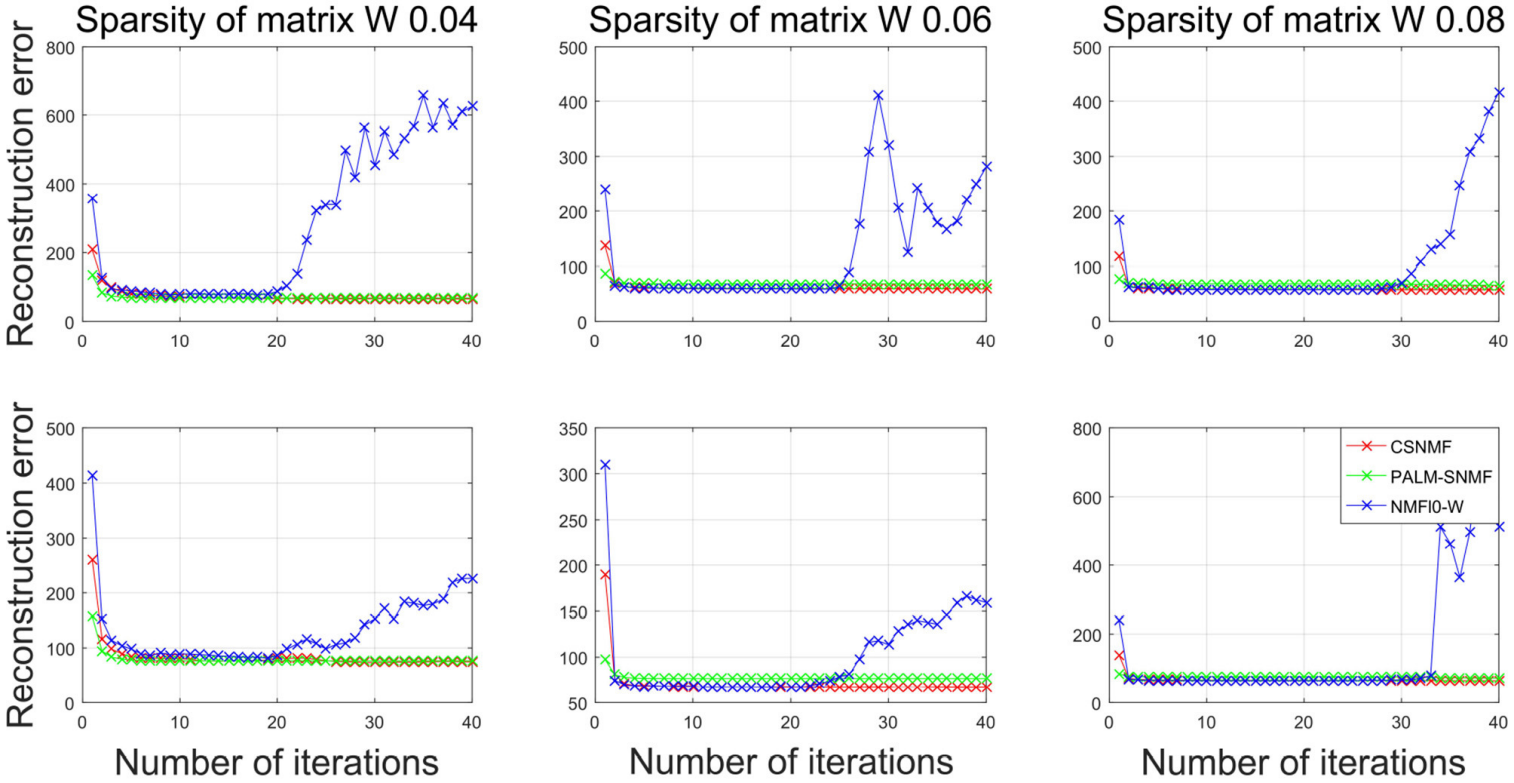
Plots of the RMSE vs. the iterations for different algorithms in neuroimaging data. The top panels corresponds to the AD patients and the bottom panels corresponds to the CN people.

Figure 10 plots the RMSE values from NMF ℓ_0_-W and PALM-SNMF to those from CSNMF vs. the number of iterations. The top three panels show the results for AD patients, and the bottom three panels show the results for CN people. Compared with NMF ℓ_0_-W, CSNMF can always derive a smaller RMSE value with convergence guarantee and the superiority is most apparent when the sparsity is lower, i.e., β = 0.04. For the NMF ℓ_0_-W approach, the RMSE increases after 20 iterations, which indicates that it is unstable and not convergent. This is might because that the matrix is extremely sparse and it is hard to derive an appropriate **W** and **H**. This also suggests that directly removing the smallest value may cause the structure of the decomposed matrix to be unstable. Compared with PALM-SNMF, CSNMF has the advantage of a rapid decrease in RMSE value. For most of the experiments, CSNMF needs less than 5 iterations to obtain a converged estimator, while PALM-SNMF needs more than 200 iterations to get a similar result. This is expected since a pseudo-Newton step is used in PALM-SNMF and our derive an optimal solution after the active set is determined. Moreover, the RMSE value of CSNMF is smaller than PALM-SNMF. In summary, our proposed CSNMF is particularly suitable for non-negative matrix factorization for such extremely sparse data such as MRI image.

**Figure 11:**
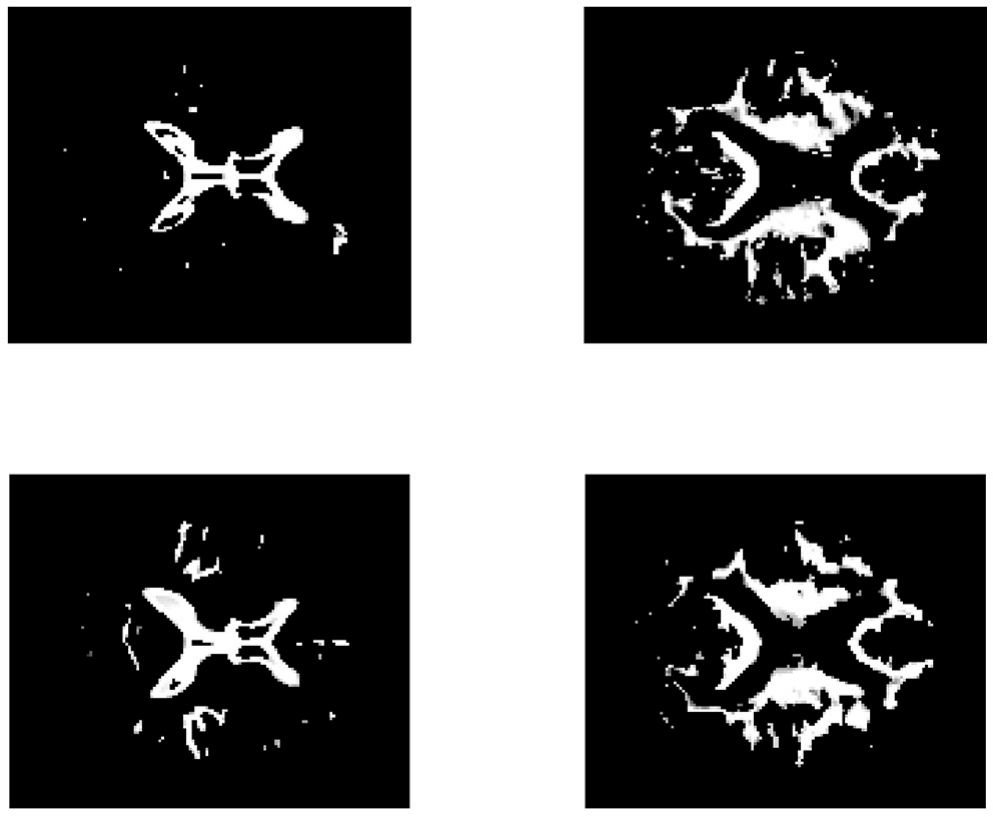
The brain feature images obtained by CSNMF. The top panels corresponds to the AD patients and the bottom panels corresponds to the CN people. From left to right, the sub-figures correspond to the same order of basis vectors.

To provide further insight into the estimated matrix, we show two basis vectors derived by the CSNMF algorithm with β = 0.04 and *K* = 15 in Figure 11. The first basis vector is actually the ventricle of brain, an important characteristic reflected by the MRI image. It can be seen that the ventricle of CN people is narrower than those of AD patients, which is consistent with the previous founding (Thompson et al., 2004). The second basis vectors describe the outline of the ventricle. For AD patients, it tends to expand upward and downward to the middle of the ventricle for the second basis vector shows a tendency to extend to the surroundings.

To further demonstrate the advantages of CSNMF in collaborative sparseness, we take out the 51st picture to form our data matrix. It is assumed that each MRI picture can be represented by a small number of sparse features, it means both **W** and **H** are sparse. In order to verify the advantages of CSNMF in collaborative sparseness, we adopt the following settings: we set *K* = 25, and 5 sets of values are adopted for β and α to achieve collaborative sparseness: (0.2, 0.8), (0.25, 0.7), (0.3, 0.6), (0.35, 0.5), (0.4, 0.4). To ensure the convergence of the results, CSNMF is executed 100 times, and PALM-SNMF is executed 5,000 times. Table 1 show the results of applying PALM-NMF and CSNMF. When the sparsity is the same, CSNMF gets smaller reconstruction error and better local representation results.

**Table 1:** Comparison of non-negative matrix factorization (NMF) PALM-SNMF and co-sparse non-negative matrix factorization (CSNMF) on Alzheimer's disease (AD) and cognitively normal (CN) (all results are timed by 10^2^).

	**Method**	**(0.2,0.8)**	**(0.25,0.7)**	**(0.3,0.6)**	**(0.35,0.5)**	**(0.4,0.4)**
AD	PALM-SNMF	7.713	7.493	7.370	7.299	7.275
AD	CSNMF	7.041	6.960	6.882	6.855	6.841
CN	PALM-SNMF	7.969	7.778	7.576	7.435	7.361
CN	CSNMF	6.951	6.876	6.844	6.784	6.811

### 4.2. Functional MRI Data

The second data set consists of resting-state cerebral fMRI (rs-fMRI), consisting of 31 subjects with AD and 32 CN people. Every subject was scanned by using 3.0 T Philips scanners. The rs-fMRI data is preprocessed using the Data Processing Assistant for Resting-State fMRI (DPARSF) software (http://rfmri.org/dpabi) based on Statistical Parametric Mapping 12 (SPM12, http://www.fil.ion.ucl.ac.uk/spm/) on the MATLAB platform (MathWorks, MA, USA). For each participant, the first 10 time points were discarded to avoid the instability of the initial MRI signals. Then, the fMRI data were corrected for the acquisition time delay and head motion. The head motion parameters of all participants were determined, and the inclusion criteria for head movement were <3.0 mm translation and <3° rotation during the fMRI scan. After these corrections, the images were directly normalized to the standard Montreal Neurological Institute (MNI) template at a 3 mm × 3 mm × 3 mm resolution. Finally, the resultant data were filtered through a temporal band-pass (0.01–0.1 Hz) to avoid the interferences of low-frequency drift and physiological noises. By using the Pearson correlation method, we obtained the FC matrix. To study the highly correlated FC brain regions with positive contributions, numbers with correlations below 0.8 are set to 0, including those with negative values. After sparse processing, the sparseness of the sparse FC matrix of normal people and AD patients are 1.78% and 2.25%, respectively. The corresponding dimensions are 8,100 × 31 and 8,100 × 32. We adopted the CSNMF method and set *K* = 15, α = 0.2 and β = 0.04, and run for 60 times to ensure convergence.

**Figure 12:**
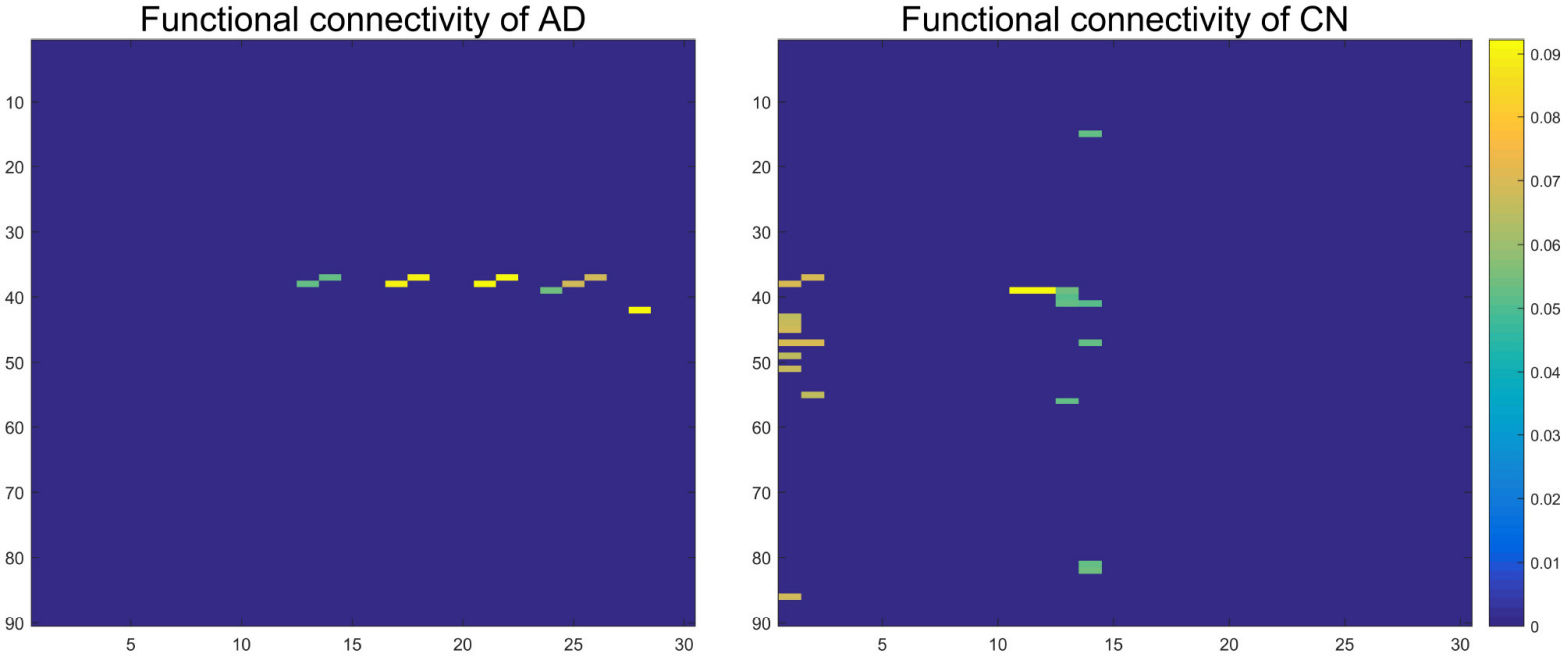
The functional connectivity between the left and right hippocampus and other brain regions. The color closer to yellow indicates the stronger functional connection.

We successfully obtained 15 basis vectors for AD patients and CN people, respectively. Each sample can be linearly represented by sparse features and coefficients. Since patients with AD are accompanied by atrophy of the hippocampus, we mainly study the FC of the hippocampus and parahippocampal gyrus. We reshape each basis vector into a 90 × 90 matrix, and take out the 37th and 38th column representing the left and right hippocampus. Finally, we get a 90 × 30 matrix about the FC of the hippocampus. Figure 12 shows the FC between the left and right hippocampus and other brain regions. The yellow part indicates a strong FC between the hippocampus and the brain area. The dark blue part has a value of 0.

**Figure 13:**
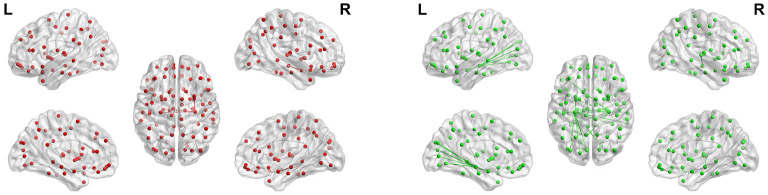
Maps of hippocampal connectivity of AD patients and CN people. The lines show significant connections between pairs of regions. The left image drawn in red is for AD patients, and the right image drawn in green is for CN people. Isolated dots indicate no connectivity.

**Figure 14:**
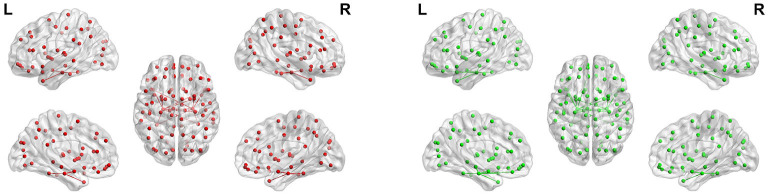
Maps of parahippocampus connectivity of AD patients and CN people. The lines show significant connections between pairs of regions. The left image drawn in red is for AD patients, and the right image drawn in green is for CN people. Isolated dots indicate no connectivity.

To show the FC of each brain region more specifically, we label according to the Anatomical Automatic Labeling (AAL) brain atlas in Figures 13, 14 of hippocampus and parahippocampus. Figure 13 shows that the connection between the left and right hippocampus of AD patients is very strong, and the right hippocampus is strongly connected with the left parahippocampal gyrus and right amygdala. However, the left and right hippocampus of normal people are connected to more brain regions except for the FC above: left orbital part of inferior frontal gyrus, right parahippocampus, left amygdala, left and right calcarineleft cuneus, left and left lingual, left superior occipital gyrus, left middle occipital gyrus, left and right fusiform gyrus, left and right superior temporal gyrus, and right middle temporal gyrus. These brain areas correspond to the symptoms of Alzheimer's patients. The lingual gyrus is a brain structure that processes vision. It is also believed to play a role in the analysis of logical conditions and encoding visual memories. Fusiform gyrus has been linked to various neurological phenomena such as synesthesia, dyslexia, and prosopagnosia.

Figure 14 shows the maps of parahippocampus connectivity of AD patients and CN people. The para hippocampal gyrus of CN people has a stronger connection than AD patients with left rectus, left hippocampal, and left superior pole temporal gyrus. Nevertheless, the FC from right parahippocampus to right inferior temporal gyrus was visibly increased in AD patients. The FC between the left and right hippocampus and the fusiform gyrus of AD patients is weakened, but the connection between the parahippocampal gyrus and the fusiform gyrus is very strong.

We found that most of the identified abnormal hippocampal FC are in AD patients with known biological interpretation. For instance, previous studies show that a connection between the hippocampus and the medial temporal lobe is existed. In medial temporal lobe, the hippocampal formation is necessary for declarative memory tasks (Small et al., 2011). Our finding also demonstrates that there is an FC between hippocampus and superiortemporal gyrus, which partly forms one of three gyri in the temporal lobe (Sun et al., 2018). It was showed that there exists abnormal FC between hippocampus and middle occipital gyrus in patients with Parkston disease (Chen et al., 2017). A previous study depicts parallel amygdalo-fusiform and hippocampo-fusiform pathways are found in normal human subjects (Smith et al., 2009). Our study also finds the same FC. As hippocampus monosynaptically connects with the orbitofrontal cortex (Small et al., 2011), the FC between hippocampus and orbital part of the inferior frontal gyrus existed in normal person cohort in our study, which is consistent with previous studies (Small et al., 2011).

## 5. Discussion

In this study, we have introduced a new co-sparse non-negative matrix factorization framework, CSNMF, for co-sparse estimation in the high-dimensional non-negative decomposition. Our CSNMF approach accurately recover the sparse basis vectors and/or the sparse coding matrix via the *l*_0_ norm constraints. Three simulations studies demonstrated that our method achieved superior accuracy in estimation and accurate identification of the non-zero elements compared with the stat-of-art methods. In real application, we applied the proposal to a MRI data from the ADNI study to get a sparse representation, and the results showed that it yields a much smaller reconstruction error. We also applied the CSNMF to the fMRI data and obtained meaningful results. Therefore, the CSNMF method is a valuable tool for non-negative matrix factorization under the high-dimensional setting.

This article represents only the first effort to derive a co-sparse non-negative matrix decomposition and there are several potential issues that should be addressed in future research. It is essential to determine an appropriate value for α and β. In previous studies on sparse NMF, there is no universal criteria for the selection of β or α. For example, Peharz and Pernkopf (2012) set three different sparsity levels of 0.10, 0.25, and 0.33 in the face experiment. Xie et al. (2017) adopted a sparsity of 0.16 when imposing *l*_0_ constraints on K-SVD. According to previous experience, for some image data like MRI images always with many non-zero elements, β = *s*_*X*_/7 (where *s*_*X*_ is the sparsity of the data matrix **X**) is recommended because it can get a small reconstruction error and obvious local feature basis. In our study, β is set to approximately equal to the sparsity of **X** while constraining the sparsity of α to 0.2 owning to the very sparse functional connection matrix. We set a smaller number for α to get a more sparse structure because the sparsity of **H** without sparsity constraints is less than 0.5.

The determination of K is also important. Specifically in the image compression process, larger K retains data information, and smaller K saves more storage space. In literature, there are several ways to determine *K* in the classical NMF problem. On intuitive method to determine an optimal *K* is choosing the one that minimizes the objective function (Paatero and Hopke, 2009). However, this method often leads to the overfitting issue as it only considers the training data. To address the overfitting problem, Yan et al. (2019) proposed a two-step cross-validation technique. Like other cross-validation techniques, it is time consuming especially when the dimensionality is too high. Similar with the total variation explained by the first *K* eigenvectors in the PCA and functional PCA methods, Brunet et al. (2004) proposed a measure called the cophenetic correlation, and selects the optimal *K* when the cophenetic correlation starts to fall. We recommend the use of cophenetic correlation in determining an optimal *K* for its simplicity and efficiency in computation.

The theoretical convergence is difficult to establish at present since the estimated error involves the alternative updating of **W** and **H**. For our proposed algorithm, it will stop when the relative estimated error is small enough. The original PDAS is shown to be converged in finite steps (Huang et al., 2018) and thus we believe our generalization of the PDAS algorithm still process this desirable convergence property. The solution of our proposed algorithm is actually a local solution, which updates only one matrix to find a coordinate-wise solution while fixing the other one. Practically, results of our several simulation and real data analysis demonstrates that the proposed algorithm does converge just like the convergence of original PDAS.

## Data Availability Statement

Publicly available datasets were analyzed in this study. This data can be found here: http://adni.loni.usc.edu/.

## Author Contributions

FW and CW proposed the algorithm and designed the experiments. HT and JC wrote the analysis of fMRI. All authors contributed to the figure preparation and critically revised the manuscript.

## Funding

CW's research is partially supported by National Science Foundation of China (12171449 and 11801540) and Natural Science Foundation of Anhui Province (BJ2040170017). HT's research is partially supported by National Key Research and Development Program of China (2018YFC1315400) and the Science and Technology Program of Guangzhou (202002030129), NSFC (72171216), and NSFC (11771462).

## Conflict of Interest

The authors declare that the research was conducted in the absence of any commercial or financial relationships that could be construed as a potential conflict of interest.

## Publisher's Note

All claims expressed in this article are solely those of the authors and do not necessarily represent those of their affiliated organizations, or those of the publisher, the editors and the reviewers. Any product that may be evaluated in this article, or claim that may be made by its manufacturer, is not guaranteed or endorsed by the publisher.
